# Respiratory health, allergies, and the farm environment: design, methods and enrollment in the observational Wisconsin Infant Study Cohort (WISC): a research proposal

**DOI:** 10.1186/s13104-019-4448-0

**Published:** 2019-07-16

**Authors:** Christine M. Seroogy, Jeffrey J. VanWormer, Brent F. Olson, Michael D. Evans, Tara Johnson, Deanna Cole, Kathrine L. Barnes, Tamara Kronenwetter Koepel, Amy Dresen, Jennifer Meece, Ronald E. Gangnon, Matthew C. Keifer, Casper G. Bendixsen, James E. Gern, the Entire WISC Study Team, Alkis Togias, Steve Sigelman, Erika von Mutius

**Affiliations:** 1 University of Wisconsin School of Medicine and Public Health, Madison, WI USA; 2 Marshfield Clinic Research Institute, National Farm Medicine Center, Marshfield, WI USA; 3 Department of Occupational Medicine, Marshfield Clinic, Marshfield, WI USA; 4 Present Address: Veterans Administration Puget Sound Healthcare System, Seattle, WA USA; 5 Division of Allergy, Immunology & Rheumatology, Department of Pediatrics, University of Wisconsin School of Medicine and Public Health, 1111 Highland Avenue, 4139 WIMR, Madison, WI 53705-2275 USA

**Keywords:** Respiratory virus, Farm, Allergies, Pregnancy, Birth cohort

## Abstract

Epidemiologic and cross-sectional studies suggest that early life farming and animal exposures are associated with major health benefits, influencing immune development and modifying the subsequent risk of allergic diseases, including asthma. The Wisconsin Infant Study Cohort (WISC) study was established in central Wisconsin to test the hypothesis that early life animal farm exposures are associated with distinct innate immune cell maturation trajectories, decreased allergen sensitization and reduced respiratory viral illness burden during the first 2 years of life. Beginning in 2013, a total of 240 families have been enrolled, 16,522 biospecimens have been collected, and 4098 questionnaires have been administered and entered into a secure database. Study endpoints include nasal respiratory virus identification and respiratory illness burden score, allergic sensitization, expression of allergic disease, and anti-viral immune response maturation and profiles. The WISC study prospective design, broad biospecimen collections, and unique US rural community will provide insights into the role of environmental exposures on early life immune maturation profiles associated with protection from allergic sensitization and significant respiratory viral disease burden. The WISC study findings will ultimately inform development of new strategies to promote resistance to severe respiratory viral illnesses and design primary prevention approaches for allergic diseases for all infants.

**Electronic supplementary material:**

The online version of this article (10.1186/s13104-019-4448-0) contains supplementary material, which is available to authorized users.

## Background

Young children under 5 years of age have a high incidence of hospitalization and morbidity caused by infections with common respiratory viruses. Respiratory infections are particularly high for infants who show early evidence of atopic diseases (e.g. atopic dermatitis) [1–3]. Epidemiologic and cross-sectional studies suggest that early life farming and animal exposures are associated with major health benefits, influencing immune development and modifying the subsequent risk of allergic diseases, including asthma [4]. Farming effects associated with decreased allergic disease include exposure to unprocessed milk (referred to as farm milk), farm animal waste and housing, and potentially bioactive substances in the farm environment [5–11]. In accordance with previously published studies in Western Europe, our study from central Wisconsin using interview and electronic health records (EHR) demonstrated that children raised in farming environments have a significant decrease in allergic diseases during the first 2 years of life [11–15]. In addition, children who live on farms in Western Europe or Wisconsin also have a marked reduction in medically-attended respiratory illnesses during the first 2 years of life compared to rural children who do not live on a farm [12, 16]. These collective findings suggest that farm-related exposures promote healthy immune system development in early life and that may lead to reduced burden of respiratory illnesses and allergic diseases. Identification and (prospective) characterization of a cohort of children that are highly protected from developing allergy disease would provide further insights and rationale in naturally-occurring environmental exposures that influence the foundational steps to prevent immune-mediated conditions.

This report details the study design, recruitment strategies, biospecimens, and unique characteristics of the Wisconsin Infant Study Cohort (WISC). Expanding upon published epidemiologic findings from our group and others, the WISC study is designed to test the hypothesis that early life animal farm exposures are associated with reduced respiratory viral illness burden, distinct innate immune cell maturation trajectories, and decreased allergen sensitization during the first 2 years of life. Findings from the WISC study will better define the environmental impact of farming-related exposures on respiratory viral illnesses, immune maturation, and allergic sensitization.

## Methods

### Design and setting

The WISC study is a prospective birth cohort of infants who are, and who are not, regularly exposed to farm environments prenatally and during the first 2 years of life. The target population included pregnant mothers from central, northern, and western Wisconsin who received prenatal and/or perinatal care from healthcare providers in the Marshfield Clinic Health System (MCHS). MCHS is a large, integrated care system serving a predominantly rural part of Wisconsin. There are approximately 3500 births per year within MCHS, which serves a region with one of the highest density of farm households (primarily dairy production) in the U.S [17]. WISC is led by the University of Wisconsin (UW) and conducted in collaboration with the Marshfield Clinic Research Institute (MCRI), which is approximately 150 miles north of the UW-Madison campus.

### Participants

Beginning in 2013, pregnant mothers from the target population were screened, invited, and enrolled in WISC. The target enrollment was 100 farm and 100 non-farm infants. Expecting mothers were enrolled prior to birth, and this included informed consent to capture study measures (described further below) on their child both before and after birth. Participants were enrolled as a dyad, as consent was signed before birth by the mother and on behalf of the child after birth. Farm mothers were defined as those who live on, or within 1/8th mile of a farm, or who work on, or have a household member who works on a farm. Farm mothers also have regular exposure to (i.e., direct personal [or household member] contact ≥ 4 days per week) with cattle (cows, calves, bulls, steers), pigs or goats. Non-farm mothers do not live on (or within 1/8th mile of) a farm, nor work on, or have a household member who works on, a farm. Non-farm mothers also do not visit a farm weekly or more, nor have any farm livestock animals as pets (e.g., cows, goats, pigs, horses, chickens). All study activities and procedures were approved by the MCHS and UW Human Subjects Institutional Review Boards.

### Recruitment and screening

Potentially eligible pregnant women were initially identified using the MCHS EHR. Non-farm eligible pregnant women are more frequent than farm eligible pregnant women, thus recruitment outreach activities were conducted at proportionate intensities to help ensure approximately equal numbers of farm and non-farm enrollments during each season. The initial enrollment area was confined to births at the Marshfield Medical Center in Marshfield, WI. In 2015, 24 additional ZIP codes in the Marshfield Epidemiologic Study Area (MESA), which encompasses 10 rural medical centers throughout north-central Wisconsin, were added to increase enrollment [18, 19]. Potentially eligible patients were first mailed an informational postcard on the study, followed by an informational letter and brochure, plus an interest response card. Once eligibility information could be verified in the EHR, an invitation letter was sent (signed by their obstetrics healthcare provider), plus up to six phone call attempts. When reached for the initial phone contact, farm or non-farm categorization and exclusion factors were again verified. If all eligibility criteria were met, a face-to-face invitation and enrollment visit was arranged, typically during scheduled prenatal visits. After informed consent forms were signed during the enrollment visit, a questionnaire to gather information about health history, environmental exposures, and lifestyle was completed.

After the birth of the participant mother’s child, a comprehensive birth record abstraction was conducted to rule out potential confounders due to complications from the pregnancy. Detailed study eligibility criteria are listed in Additional file 1: Table S1. Exclusion criteria are: (1) maternal use of antibiotics (except Group B Strep prophylaxis) or corticosteroids in the last trimester of pregnancy; (2) delivery at ≤ 34 weeks gestation; (3) perinatal infections or prolonged rupture of membranes; (4) significant congenital anomalies; (5) significant respiratory distress after delivery.

### Study visits

As outlined in Table 1, in-person visits occur prenatally and when the infant is at 2, 9, 12, 18, and 24 months of age (with a permitted window of − 1 to + 3 months for each study time point). All visits are led by a trained Research Coordinator and occur at participants’ homes or coincide with a scheduled well child healthcare visit at their clinic. In addition, after the infant is born, mothers are contacted by telephone every 3 months to complete a study questionnaire. Prompts are sent by telephone or mail at monthly intervals to schedule study activities or remind about upcoming study procedures.

**Table 1:** WISC study activity schedule

Activity	Screen	Prenatal	Birth	2 months	6 months	9 months	12 months	15, 18, 21 months	24 months	Illness (as needed)
Eligibility	X									
Consent		X								
Medical record extraction			X	X		X			X	
Respiratory illness symptom diary										X
Questionnaires										
Prenatal		X								
Postnatal				X		X			X	
Infant feeding and diet				X	X	X	X		X	
Child diet								X	X	
Respiratory allergy				X	X	X	X	X	X	
Occupational exposures									X	
Personal samples										
Vaginal swab		X								
Blood collection			X^a^				X		X	
Nasal swab				X		X	X	X^b^	X	
Stool				X		X			X	
Skin				X		X			X	
Saliva				X		X			X	
Urine				X		X			X	
Breast milk				X						
Nasal swab (viral)										X
Nasal brushing									X	
Environmental samples										
Dust (settled (incl barn dust, if applicable)				X		X			X	
Dust (vacuum)				X						
Drinking water				X		X			X	
Farm milk						X			X	

^a^Cord; ^b^18 months only

### Endpoints and outcomes

**Fig. 1:**
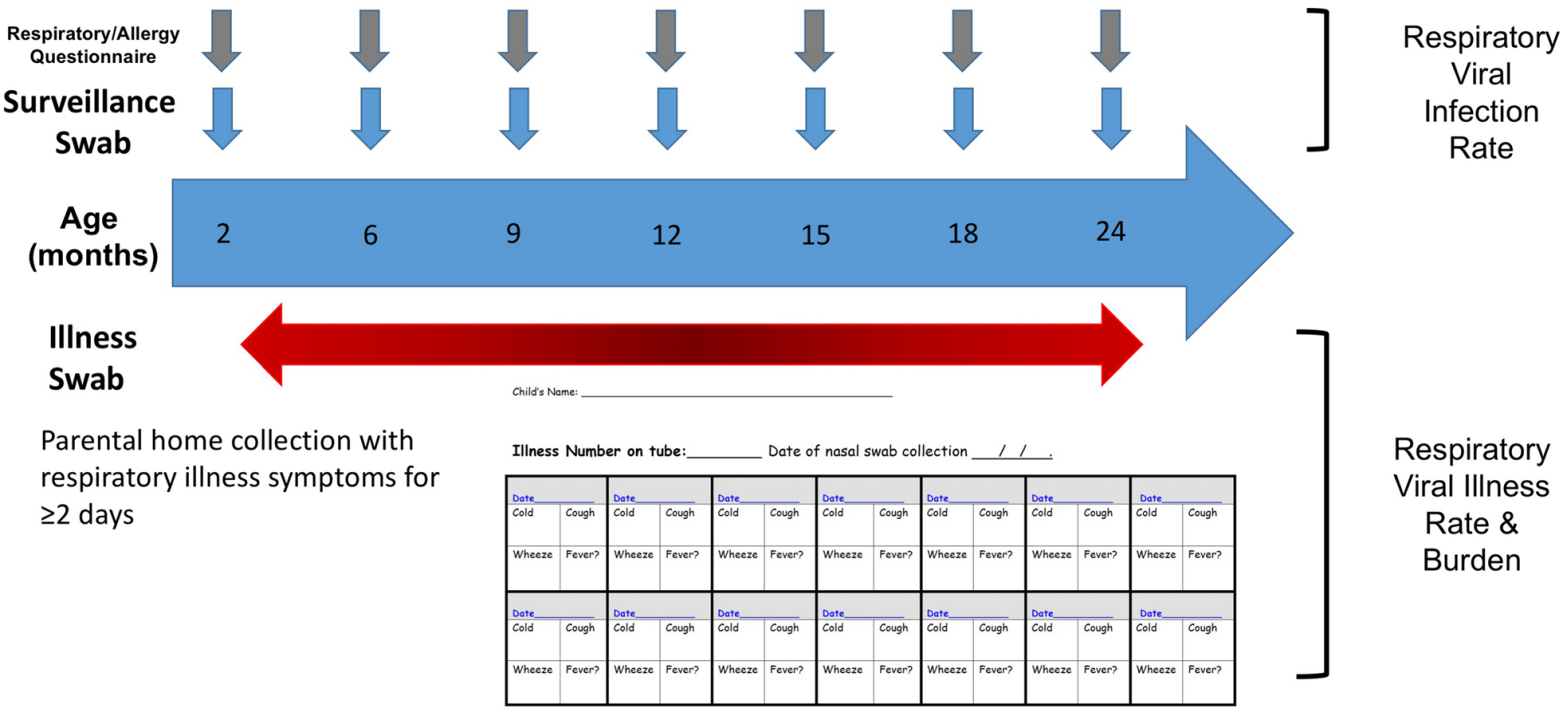
Schematic of biospecimen and data collection for WISC study primary outcome. Middle turbinate nasal swabs are collected using FLOQSwabs™

The primary outcome is respiratory viral illness burden from 2 months to 2 years of age (Fig. 1). Endpoints analyzed for this outcome include nasal swab respiratory virus detection and respiratory illness burden index. A respiratory illness is defined as at least 2 consecutive days of cold, cough, or wheeze. Nasal swabs collected at routine surveillance timepoints and during illness episodes are assayed for all common respiratory viruses (e.g. rhinovirus, respiratory syncytial virus, coronavirus, influenza, parainfluenza, metapneumovirus, bocavirus, enterovirus and adenovirus) by multiplex reverse transcription polymerase chain reaction (RT-PCR) at the UW study site [20]. Rhinovirus isolates are partially sequenced to identify strains and to differentiate lengthy single infections from serial infections with different rhinovirus strains. During illnesses, parents are asked to record daily information consisting of scored illness signs/symptoms and temperature measurements (if fever present) in calendar-formatted paper diaries. Illness signs/symptoms are recorded on a 4-point scale (none, mild, moderate, severe) similar to previously published studies [21–23]. Parents or legal guardians record the data every day until symptoms resolve. The respiratory illness burden index is calculated as area under the curve (AUC) for symptom scores and days of illness. Linked EHR data is also used to capture medically-attended respiratory illnesses via diagnostic codes and/or respiratory antiviral medication prescriptions.

Secondary outcomes include allergic sensitization, expression of allergic disease, and antiviral innate immune cell maturation. For the allergic sensitization outcome, plasma IgE-specific antibodies are analyzed at several time points using a two-tiered approach with multiscreen panels (foods and environmental airborne allergens [Phadiatop, Waltham MA]) and, where indicated, individual IgE-specific quantitative measurements. Atopic dermatitis (incidence, cumulative prevalence, and resolution) is defined as parental report of chronic pruritic skin rash or doctor-diagnosed atopic dermatitis on the EHR.

To determine antiviral immune responses, we have adapted and optimized a previously published high-throughput assay stimulation platform and multi-parameter flow cytometry panel [24]. Standardized assay plates with TLR agonists (R848 [TLR7/8]; LPS [TLR4]; CpGA [TLR9]) and infectious rhinovirus-A16 were preformatted for blood sample processing and stimulation at the MCRI study site. Collected blood samples are processed and stimulated within 24 h of collection. Pilot validation studies showed comparable assay read-outs within this processing time criteria (Additional file 1: Figure S1). Sample staining, acquisition, and analysis are performed at the UW study site. Precision testing demonstrated excellent inter-assay and intra-assay performance with an average assay coefficient of variation 14.3% (range for various agonists: 6.5–27.3%, Additional file 1: Figures S2, S3, respectively). As an exploratory endpoint, T regulatory (Treg) cells were identified using immune phenotyping and epigenetic analysis using previously published approaches [25].

### Sample size

The primary outcome measures for WISC are (1) rate of respiratory viral infections at scheduled quarterly visits and (2) respiratory illness burden index for the first 2 years of life. For the first outcome, the proposed sample size of 100 participants per group with 10% dropout will provide 80% power to detect a reduction in infection rate to 30% in the farm participants compared with 40% in the non-farm participants (odds ratio of 0.64) using a two-sided 5% level test, conservatively assuming that a correlation of 0.5 between occurrence of respiratory infections at two visits within the same child. Using data from the Childhood Origins of AsThma (COAST) birth cohort (yielding an estimated standard deviation for the respiratory illness burden index over the first 2 years of life of 32.6) and published epidemiologic farm studies, the proposed sample size of 100 farm and 100 non-farm participants with an expected retention rate of 0.9 will provide at least 80% power to detect a 19% reduction in respiratory illness burden index in farm participants (corresponding to a mean respiratory illness burden index of 65.4 in farm infants compared to 78 in non-farm infants) using a two-sided 5% level test [12, 26, 27].

### Covariates

Several previously validated questionnaires were used in the WISC study (Table 2). Questionnaires are administered face-to-face by Research Coordinators or over the phone, depending on visit schedule and participant preferences. A Research Electronic Data CAPture (REDCap™) database was developed for questionnaire data entry and transcription of other EHR-extracted study variables [28]. Research Coordinators record participants’ responses on hard copy forms of the questionnaires and re-enter survey data in REDCap™ upon their return to the office. If the questionnaire is administered over the phone, the data is direct-entered. Regular quality assurance, including (blinded) double data entry on randomly selected questionnaires, is conducted to ensure data integrity. Other information such as medication use and acute care episodes are extracted from the EHR. The birth record containing information on the newborn child and mother is also extracted to record relevant study details related to eligibility. Other information includes infant’s, mother’s, and father’s demographic characteristics, noted birth complications, vaccine administration in the prenatal period, mother’s chronic disease status, anesthesia use, method of delivery, and medication use of mothers during pregnancy and while breastfeeding.

**Table 2:** WISC study questionnaires

Instrument name	Purpose	References
Prenatal	Family health history Environmental exposures Lifestyle	Gabriela [7]
Postnatal	Environmental exposures Lifestyle	Gabriela [7]
Infant feeding and diet	Maternal dietary history Infant dietary history	2000 NCI Multifactor Screener (https://epi.grants.cancer.gov/nhis/multifactor/)
Child diet	Child dietary history	n/a
Respiratory and allergy	Allergies Respiratory illnesses Health care utilization	ISAAC [42]
Occupational exposures	Farm exposure Occupational-related respiratory hazards	Adapted from [43]
Medication use	Mother until cease breastfeeding Infant/child	n/a

*n/a* not applicable

### Biospecimen processing and tracking

A number of environmental and personal biospecimens are collected. A study ID number is randomly assigned to each participant and linked to laboratory and study metadata. WISC study data management, including data validation, storage and quality assurance, is conducted by MCRI staff. Data files are transferred between study sites via an honest broker and secure, password-protected File Transfer Protocol. Biospecimens are collected for defined study outcomes and additional biospecimens are being collected for future analyses and endpoints (Table 3). For uniformity in data collection, detailed standard operating procedures and data collection instruments were developed for WISC (available upon request). To control for biospecimen collection variances, lot tracking of all collection materials is documented and blank collection tubes from each lot are saved for all microbiome-related biospecimens. Biospecimens were collected during scheduled clinic visits, in the home by trained Research Coordinators, or by the participating child’s parent or legal guardian in the home. Viral nasal swab biospecimens are collected during scheduled time points and during respiratory illnesses at home or in the clinic by parent/legal guardians and trained Research Coordinators. After collection, swabs are placed in transport medium and mailed to MCRI. Specimens are then frozen and stored, pending further processing and analysis at the UW study site. Viral diagnostics are conducted as previously described [29].

**Table 3:** Biospecimen type and testing plan

Sample type	Testing plan
Maternal vaginal swab	Vaginal microbiome
Maternal breast milk	sIgA, milk microbiome, metabolomics
Infant/child blood	Innate immune cell function, Treg cell profile, plasma lipidomics, allergen-specific and total IgE, cryopreservation
Infant/child nasal swab	Nasal microbiome, respiratory virus detection (surveillance)
Infant/child nasal illness swab	Respiratory virus detection (illness)
Child nasal brushing^a^	Transcriptomics
Infant/child stool	Gastrointestinal microbiome
Infant/child urine	Metabolomics
Infant/child skin swab	Skin microbiome
Infant/child saliva	Oral microbiome
Household airborne and vacuum dust	Environmental microbiome (bacterial and fungal)
Drinking water	Environmental microbiome
Barn airborne dust	Environmental microbiome (bacterial and fungal)
Farm milk	Microbiome, metabolomics

*sIgA* secreted immunoglobulin A, *Treg* T regulatory cell

^a^Obtained in subset of participants

### Analyses

Categorical data were compared between farm and non-farm using the Chi squared test for association.

Percentages were rounded to the nearest whole number.

## Results

### Enrollment

**Fig. 2:**
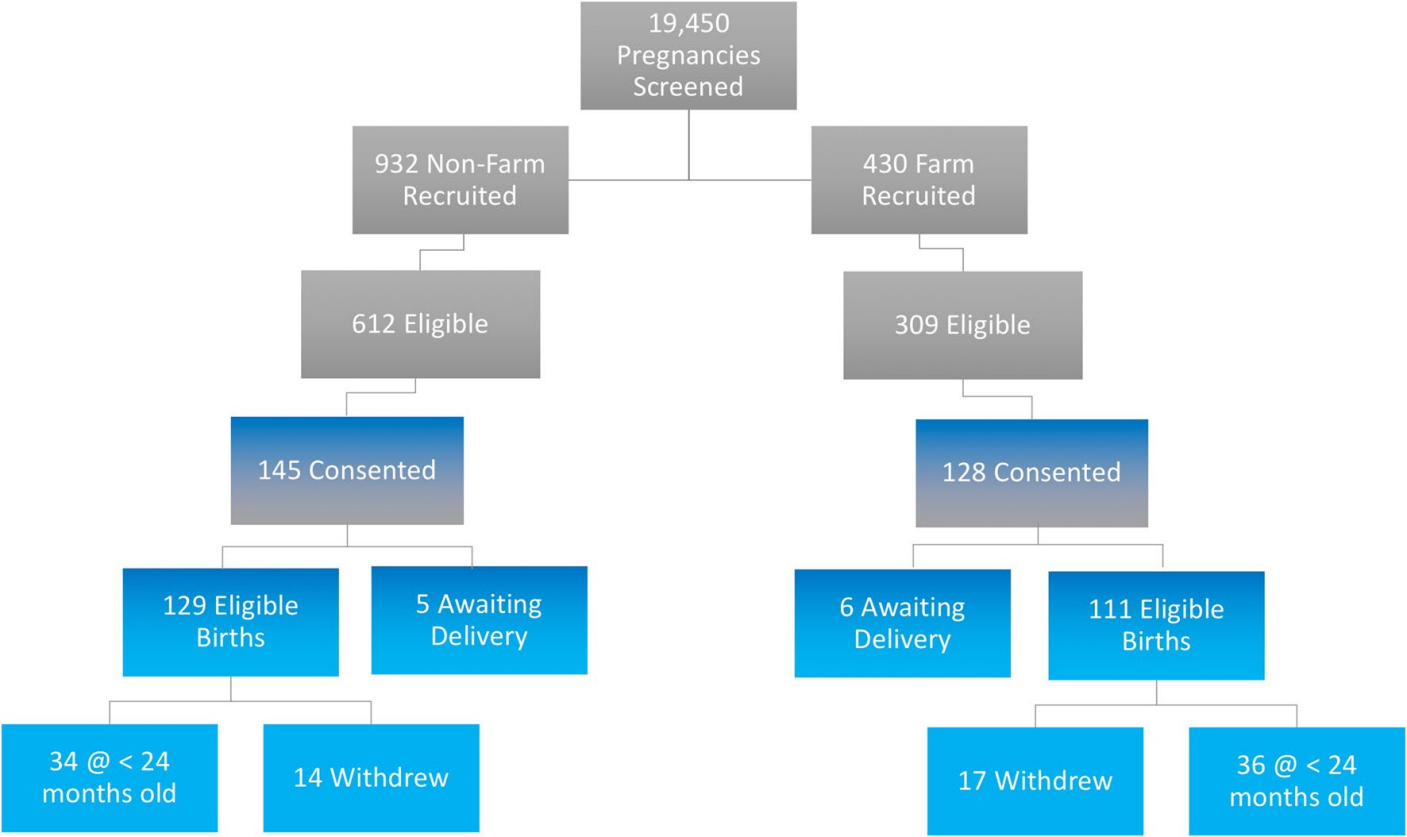
WISC study recruitment and enrollment

As outlined in Fig. 2, both the farm and non-farm cohorts of the WISC study have been fully enrolled. During the ~ 5-year timeframe from April 2013 through May 2018, the total number of pregnancies electronically screened was 19,450. The majority of these women (67%) resided outside the study’s geographic catchment area. Another 18% of those screened were too far along in their pregnancy to begin recruitment, 1% had exclusionary medical conditions, and 8% could not be reached for study contact. The remaining 6% made up the study recruitment pool, consisting of 932 potential non-farm and 429 potential farm women. After initial contact, 612 non-farm and 309 farm women were found to be eligible for WISC. Reasons for ineligibility at this stage mostly included additional medical issues, and ambiguity in establishing farm vs. non-farm status. Of the eligible non-farm women, 145 (24%) consented to WISC, and of eligible farm women, 128 (41%) consented. The enrollment rate was significantly higher for farm vs. non-farm mothers ($X^{2}_{{\left( {{\text{DF}} = 1} \right)}}$ = 30.95, *p *< 0.001). After birth, 8% of non-farm and 9% of farm children were found to be ineligible due to medical complications from the pregnancy. To date, 115 non-farm families and 94 farm families are enrolled in the study. Of these, 12% have withdrawn, and 4% are still awaiting delivery. The median follow-up time for enrolled children is 27 months. Additional families will be enrolled to replace those withdrawn from the study.

Sociodemographic characteristics of the farm and non-farm groups were similar, except that there were significantly more male children born in the farm group (p = 0.03, Table 4). Most enrollees lived in counties near the main study site in Marshfield. The vast majority of enrolled mothers gave birth between the age of 25–34 years, and nearly two-thirds had a college education. Home environment and personal health characteristics are described in Table 5. Compared to non-farm mothers, a significantly greater proportion of farm mothers owned a dog (p < 0.001) or cat (p < 0.001), and regularly drank farm milk (i.e., unprocessed milk obtained directly from a farm) during their pregnancy (p < 0.001), A significantly greater proportion of farm children (at age 2 months) spent at least 1 day per week at another home (p = 0.02), and non-farm mothers were more likely to work outside the home (p = 0.005). Study covariates that were not significantly different between the groups include mode of delivery, maternal history of varied allergic diseases, maternal smoking, and exclusive breastfeeding during the first 2 months of life.

**Table 4:** Baseline sociodemographic characteristics of mothers and children enrolled in the wisc study, stratified by farm status

Characteristic	Farm (n = 111) (%)	Non-farm (n = 129) (%)	p-value
Mother			
Maternal age (years)			NS
≥ 40	2	2	
35–39	20	9	
30–34	40	44	
25–29	32	40	
18–24	7	5	
Marital status			NS
Married or living with a partner	89	88	
Single	5	8	
Unknown	6	4	
Education			NS
High school or less	6	6	
Associate degree or some college	29	28	
Bachelor’s degree	50	45	
Graduate degree	11	18	
Unknown	4	3	
Annual household income			NS
≥ $100,000	18	22	
$25,000–$99,999	63	68	
< $25,000	9	4	
Unknown	10	6	
Health insurance			NS
Private	37	46	
Public-assisted	60	53	
Unknown	3	1	
Children			
Enrollment year			NS
2013	5	3	
2014	16	13	
2015	37	41	
2016	23	29	
2017	13	6	
2018	6	8	
Season of birth			NS
Winter (Dec–Feb)	23	22	
Spring (Mar–May)	28	25	
Summer (Jun–Aug)	22	27	
Fall (Sep–Nov)	27	26	
Sex			*0.03*
Female	43	58	
Male	57	42	
Race/ethnicity			NS
White	99	94	
Black or African American	1	2	
Asian	0	2	
Other	0	2	
County of residence			*<* *0.0001*
Wood	14	68	
Clark	23	17	
Marathon	23	19	
Chippewa	9	0	
Barron	8	0	
Other (13 counties)^a^	23	5	

Values are reported as frequency (% of group total)

A *p* value ≤ 0.05 (in italics) was considered significant

*NS* not significant

^a^Study residence ≤ 5% in either group were combined under other

**Table 5:** Home environment and personal health characteristics of WISC study participants

Characteristic	Farm (n = 111) (%)	Non-farm (n = 129) (%)	p-value
Number of children in household			NS
≥ 4	19	12	
3	23	15	
2	26	42	
1	25	23	
Unknown	7	8	
Employment (mother)			*0.005*
Employed outside home/farm	60	78	
Not employed outside home/farm	36	19	
Unknown	4	3	
Mode of delivery			
Vaginal	83	79	NS
C-section	17	21	NS
Child exclusively breastmilk fed (2 months)	50	47	NS
Child spends time at least 1 day per week (2 month infant)			
Daycare facility	14	21	NS
Another home	46	31	*0.02*
Maternal smoking			
During year prior to pregnancy	2	4	NS
During pregnancy	9	15	NS
Maternal regular^a^ farm milk consumption during pregnancy	16	2	*<* *0.0001*
Dog ownership (prenatal)^b^	73	52	*0.0009*
Dog spends time indoors	31	35	NS
Cat ownership (prenatal)^b^	76	32	*<* *0.0001*
Cat spends time indoors	19	15	NS
Maternal history of allergic rhinitis (ever)	11	18	NS
Maternal history of asthma (ever)	16	21	NS
Maternal history of atopic dermatitis (ever)	18	20	NS

Values are reported as frequency (% of group total)

A *p* value ≤ 0.05 (in italics) was considered significant

*NS* not significant

^a^Regular is defined as weekly or greater frequency

^b^5% missing data or refused

Farm characteristics and related activities of farm group participants are listed in Table 6. The WISC study farm group eligibility criteria was designed to enroll pregnant woman exposed to livestock farming with an emphasis on dairy cows, along with regular contact between the pregnant mother and livestock. A subset of the farm pregnant woman (16%) worked on the farm but did not live there. For the pregnant women that live on a farm, 31% also report working on the farm. The WISC farm group mostly live on dairy farms (77%), and 50% are small dairy farms (≤ 100 cows per farm, Table 6 and Additional file 1: Figure S4). Half of the farms have more than one type of farm animal and the vast majority of WISC farms also grow crops. During pregnancy, most of the farm women had regular direct contact with cattle, hay, straw, feed grain, and silage. The majority of both 2 and 9 month old farm group infants spent time in the animal barns on a daily or weekly basis.

**Table 6:** Farm characteristics of WISC mothers/infants in the farm group

Characteristic/activity (n = 111 unless otherwise stated)	
Farm residence and work status	
Live/work on farm	80%
Work only on farm	16%
Unknown	4%
Animals kept on farm	
Cows	77%
Cattle (bulls, steers)	32%
Goats	13%
Pigs	19%
Poultry	32%
Horses	13%
Sheep	6%
Other	11%
Number of farm animal species	
1	43%
2	25%
3	14%
4	8%
5	2%
6	1%
Unknown	7%
Crops grown and harvested	88%
Mother: regular^a^ direct contact during pregnancy with	
Cattle (cows, calves, bulls, steers)	66%
Goats	7%
Pigs	10%
Poultry	25%
Hay	76%
Straw	63%
Feed grain	66%
Silage	58%
Manure	30%
Unknown	5%
2 month infant (n = 101)	
Regular exposure to cattle	58%
Regular exposure to goats	5%
Regular exposure to pigs	8%
Regular exposure to poultry	11%
Regular exposure to forage^b^	35%
Regular farm milk ingestion	1%
9 month infant (n = 89)	
Regular exposure to cattle	50%
Regular exposure to goats	9%
Regular exposure to pigs	9%
Regular exposure to poultry	14%
Regular exposure to forage	44%
Regular farm milk ingestion	2%

^a^Regular is defined as weekly or greater frequency

^b^Forage is defined as hay, haylage, or silage

### Biospecimen collection

**Fig. 3:**
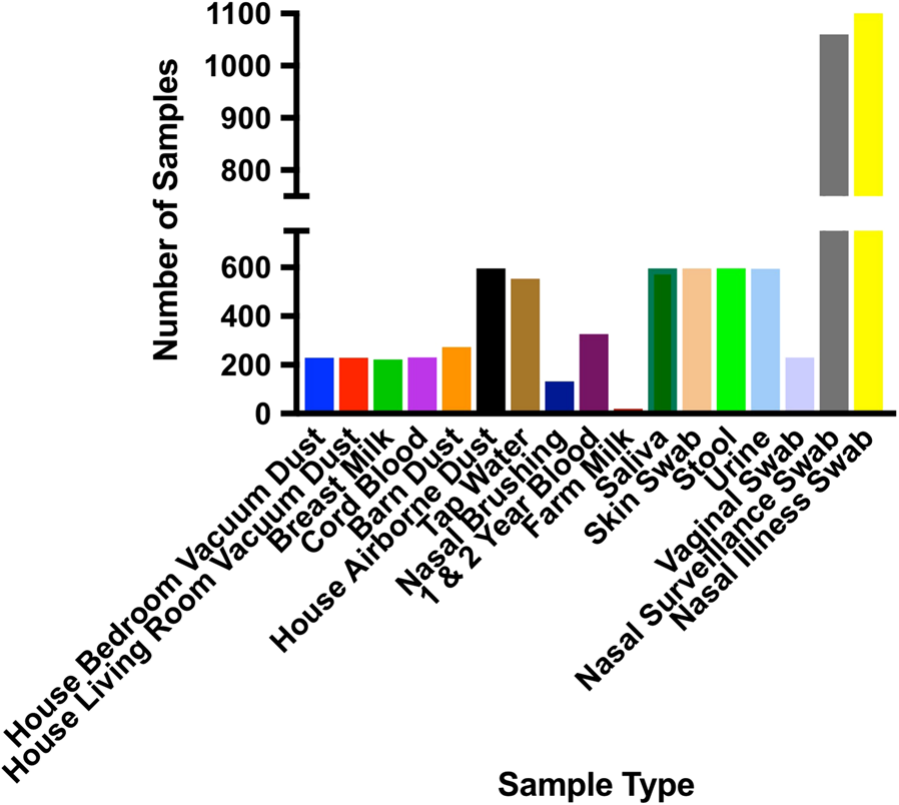
Number and type of biospecimens collected to date for the WISC study

To date, 1999 nasal swabs have been collected. This includes 1105 nasal illness swabs from both farm and non-farm participants. The WISC study has collected 197 cord blood samples and 267 1- or 2-year blood samples for immune studies. In addition to the immune and viral studies, the WISC study has collected a large number of biospecimens to define group-specific patterns of microbial exposures and colonization, and for future analysis of gene expression, proteins and metabolites. As of May 2018, a cumulative total of 16,522 biospecimens have been collected for the WISC study (Fig. 3).

## Discussion

The WISC study is the first rural and farming birth cohort in the US. There are several birth cohorts in the United States established to understand the origins of allergic diseases and role of viral respiratory infections, but none focus on farm exposures early in life [30–32]. Identification and in-depth characterization of population subgroups with strong protection from allergic disease are necessary to better define the immune mechanisms of allergic disease inception and inform rationale prevention strategies.

The WISC study has reached its enrollment goal. Baseline characteristics appeared reasonably balanced between farm and non-farm groups, though the participation rate is higher in farm families. Reasons for this are not understood, but could involve an opportunity to participate in a study that may reflect favorably on farming lifestyles. In general, WISC participants had a higher education level and included about twice as many college graduates as is typically seen in Wisconsin adults [33].

The WISC study comprehensive biospecimen collections and excellent participant retention to date will allow us to not only adequately test the WISC study hypothesis, but will also provide opportunities for additional studies. For example, specimens are being collected to enable future studies of associations between serum and airway microbes and metabolites and study outcomes. Notably, the Wisconsin diary industry was founded by Bavarians, and about 85% of the Wisconsin farmers have Bavarian surnames. Comparisons of findings in WISC and Western European cohorts such as the Protection against Allergy—Study in Rural Environments (PASTURE) study should be of great interest [34]. For example, WISC farm mothers reported a considerably lower rate of farm milk ingestion relative to European farm mothers [35].

Methodological strengths of the WISC study include recruitment from a well-defined source population, directly observed exposure classifications, and laboratory-confirmed assessment of key illness and allergy outcomes. The WISC study includes families with broad range of farm exposures and practices, and this presents opportunities to compare quantitative and qualitative measures of farm exposures to study outcomes.

In addition to the ongoing data collection and analysis to address study outcomes during the first 2 years of life, the WISC study has now been extended to follow the participants up to 8 years of age. The WISC study is also part of the recently established Children’s Respiratory and Environmental Workgroup (CREW) Consortium, funded by the Environmental Influences on Child Health Outcomes (ECHO) program. ECHO-CREW aims to harmonize data from 12 US birth cohorts focused on asthma inception, risk factors, and causal pathways. Inclusion of the WISC study cohort, the only rural and farming cohort, alongside urban, suburban, and varied geographic and race/ethnicity will provide a broader representation of US demographics.

## Conclusions

The WISC study is a rural and farming birth cohort unique to the US. Our study design and successful enrollment provide a solid foundation to begin addressing our study outcomes. The findings from the WISC study will provide the first prospective, serial analysis of viral respiratory disease burden, immune development, and allergen sensitization with varied environmental exposures. This knowledge will enable development of safe and novel strategies to prevent respiratory diseases in the general population, monitor response to immunotherapies, and predict individual risk for allergic disease.

## Limitations

Common to most longitudinal studies, attrition over time is a potential concern as enrollees age and/or move out of the study area. Also, as is typical in the rural Midwest, the sample lacks racial diversity and, while adequately sized for main outcome analyses, may be small to test for effect modification in select subgroups [36]. A potential limitation of our study is the lack of air quality monitoring, particularly since ambient air pollution exposure in early life has been associated with the inception of allergic diseases and respiratory health [37–41]. This limitation could be overcome though accessing open source regulatory monitoring data.

## Additional file

**Additional file 1: Table S1.** Detailed Inclusion and Exclusion Criteria. **Figure S1.** Effect of Processing time on assay read-outs. **Figure S2.** Inter-assay variability. **Figure S3.** Intra-assay variability. **Figure S4.** WISC Study farm group dairy cow numbers.

## Data Availability

The WISC study detailed manual of procedures and specific standard operating procedure source documents are available from the corresponding author on reasonable request.

## Abbreviations

AUC: area under the curve
CREW: Children’s Respiratory and Environmental Workgroup
COAST: Childhood Origins of AsThma
ECHO: Environmental Influences on Child Health Outcomes
EHR: electronic health records
MCHS: Marshfield Clinic Health System
MCRI: Marshfield Clinic Research Institute
MESA: Marshfield Epidemiologic Study Area
PASTURE: Protection against Allergy—Study in Rural Environments
REDCap™: Research Electronic Data CAPture
RT-PCR: reverse transcription polymerase chain reaction
Treg: T regulatory
UW: University of Wisconsin
WISC: Wisconsin Infant Study Cohort
