# Supplementary material for: Respiratory health, allergies, and the farm environment: design, methods and enrollment in the observational Wisconsin Infant Study Cohort (WISC): a research proposal
Source: BMC Res Notes. 2019 Jul 16;12:423. doi: 10.1186/s13104-019-4448-0 (PMC6636141; doi:10.1186/s13104-019-4448-0)
Supplement: Supplementary file 1 — Additional file 1: Table S1. Detailed Inclusion and Exclusion Criteria. Figure S1. Effect of Processing time on assay read-outs. Figure S2. Inter-assay variability. Figure S3. Intra-assay variability. Figure S4. WISC Study farm group dairy cow numbers. [file 13104_2019_4448_MOESM1_ESM.docx]

**Table S1.** Detailed Inclusion and Exclusion Criteria

|  | **Inclusion Criteria** | **Exclusion Criteria** |
| --- | --- | --- |
| For the child | 1. Subject’s parent or legal guardian must be able to read, understand and provide informed consent (child must be able to provide assent at age 8)  2. Healthy neonates ≥34weeks gestation | 1. Inability or unwillingness of subject’s parent or legal guardian to give written informed consent or comply with study protocol  2. Preterm deliveries <34 weeks gestation |
| For the mother | 1. Receives prenatal care in Wisconsin. Farm inclusion: farm with cattle, cows or goats. If it is a cattle farm, it is preferred that the participant is exposed to the cattle. Pig-only farms or farms with pigs and other livestock excluding cattle, cows or goats may be enrolled under the PI’s discretion.  2. Age of at least 18 yrs | 1. Perinatal infections  2. Use of antibiotics (except Group B strep prophylaxis) or corticosteroids in the last month prior to delivery,  3. Chronic medical conditions (including but not limited to: HIV infection, autoimmune disease requiring chronic medication)  4. Past or current medical problems or findings from physical examination or laboratory testing that are not listed above, which, in the judgment of the study investigators, may pose additional risks from participation in the study, may interfere with the participant’s ability to comply with study requirements or that may impact the quality or interpretation of the data obtained from the study.  5. Works or resides on farm without cattle, cows, pigs or goats, but instead with horses, sheep or poultry ie. sheep-only farms  6. Works or resides in household with pet farm animals.  7. Age of <18 yrs.  8. Resides in nonfarm household < 1/8 mile from animal farm. |
| For both | 1. Residence in Wisconsin | 1. Residence in Wisconsin |

Figure S1: Effect of Processing time on assay read-outs. pDC function after CpGA stimulation. Blood samples collected in two separate sodium heparin tubes from 3 different deidentified adult subjects. One tube was processed immediately and the second tube was kept at room temperature for 24 hours after collection and then processed and stimulated in an identical manner.

Figure S2 : Inter-assay variability. Each run is same day, same sample and two trained technicians. The blood sample is divided between the two trained technicians for parallel processing and stimulation at the MCRF site. Sample acquisition and analysis was performed by one technician at the UW site.

Figure S3: Intra-assay variability. LPS stimulated peripheral blood mononuclear cells from three deidentified adult subjects. Average %CV 7.59

Figure S4: WISC Study farm group dairy cow numbers. N=111 (missing data on n=8 [7%]).
